# The Clinical Impact of the Decipher Genomic Classifier in Prostate Cancer

**DOI:** 10.5152/eurasianjmed.2025.25828

**Published:** 2025-05-05

**Authors:** Sophia Li, Stephanie A. Berg, Mutlay Sayan

**Affiliations:** 1 Department of Radiation Oncology, Brigham and Women’s Hospital and Dana Farber Cancer Institute, Harvard Medical School, Boston, MA, USA; 2 Lank Center for Genitourinary Oncology at Dana-Farber Cancer Institute, Harvard Medical School, Boston, MA, USA

**Keywords:** Prostate cancer, genomic classifier, biomarkers, risk stratification, precision medicine

## Abstract

The Decipher genomic classifier (GC) is a 22-gene expression test that refines risk stratification and informs treatment decisions in localized prostate cancer. Traditional clinicopathologic factors, including prostate-specific antigen levels and kinetics, Gleason score, histologic variants, and tumor stage, do not fully capture disease heterogeneity, leading to potential overtreatment or undertreatment. The Decipher GC has demonstrated clinical utility across risk groups, helping to distinguish candidates for active surveillance in low-risk prostate cancer, refine the need for androgen deprivation therapy in intermediate-risk disease, and guide treatment intensification in high-risk patients. In the post-radical prostatectomy setting, the GC aids in determining the need for early salvage radiation therapy and hormonal therapy. While retrospective studies support its prognostic value, limitations include heterogeneity in study designs and the lack of established predictive utility for treatment response. Ongoing prospective trials, such as NRG GU-009 and NRG GU-010, aim to validate further the Decipher GC’s role in clinical decision-making and treatment personalization.

**Main Points**

- Decipher GC improves risk stratification in prostate cancer by providing independent prognostic information beyond traditional clinicopathologic factors.
- Decipher GC guides treatment decisions across all prostate cancer risk groups by identifying low-risk patients for surveillance, refining therapy in intermediate- and high-risk diseases, and informing post-prostatectomy management.
- Ongoing prospective trials, such as NRG GU-009 and NRG GU-010, will further validate Decipher’s role in real-time clinical decision-making, addressing current limitations in predictive utility.

## Introduction

Prostate cancer is the second most common malignancy in men worldwide, with an estimated 1.5 million new cases diagnosed and nearly 400 000 deaths globally in 2022.^1^ The clinical behavior of prostate cancer varies widely, with some cases managed conservatively while others require multimodal treatment.^2^^-^^4^ Risk stratification plays a crucial role in guiding treatment decisions and has traditionally relied on prostate-specific antigen (PSA) levels, Gleason score, tumor stage, and the percentage of positive biopsy cores.^5^^-^^7^ The National Comprehensive Cancer Network (NCCN) risk stratification framework further refines patient classification into low-, intermediate-, and high-risk groups, influencing management strategies such as active surveillance, radical prostatectomy (RP), radiation therapy (RT), and androgen deprivation therapy (ADT).^2^ However, clinicopathologic factors alone fail to capture the biological heterogeneity of prostate cancer, leading to both overtreatment in some cases and undertreatment in others.

Advancements in molecular oncology and genomic profiling have enabled the integration of prognostic and predictive biomarkers to refine risk stratification and personalize treatment strategies. Several tissue-based molecular tests, including Ki-67 immunohistochemistry,^8^^-^^10^ cell cycle progression score (Prolaris),^11^^-^^15^ genomic prostate score (Oncotype DX),^16^^-19^ and 22-gene genomic classifier (Decipher),^20^^-^^22^ have been developed to provide additional prognostic information beyond conventional clinicopathologic factors. Among these tests, the Decipher genomic classifier (GC) has emerged as the most widely used molecular prognostic test in clinical practice. It utilizes whole transcriptome RNA expression profiling to assess the risk of metastasis, biochemical recurrence (BCR), and prostate cancer-specific mortality (PCSM).^23^ Originally developed for post-RP patients to predict disease progression, it has since demonstrated utility across multiple clinical settings, including localized disease, post-RP BCR, and high-risk prostate cancer requiring treatment intensification.^24^ ^25^ This review focuses on the impact of the 22-gene GC on prostate cancer treatment decision-making, examining its role in low-, intermediate-, and high-risk prostate cancer, as well as its influence on post-RP management.

## Impact on Treatment Decision-Making

### Low-Risk Prostate Cancer

For patients with low-risk prostate cancer, the primary challenge is distinguishing those who can safely remain on active surveillance from those who may require early intervention. Traditional risk assessment based on PSA levels, Gleason score, and clinical staging often lacks the precision to fully capture disease biology. The Decipher GC provides additional molecular insights that refine risk stratification, helping to identify patients who may be ideal candidates for long-term surveillance. Prospective evidence from the Michigan Urological Surgery Improvement Collaborative demonstrated that patients with low GC scores (<0.45) remained on active surveillance for more than twice as long as those with high-risk scores, with a median delay of 19.4 months before requiring treatment.^26^ Similarly, data by Kim et al^27^ showed that patients with the lowest GC scores had a high negative predictive value for adverse pathology at RP, reaching 96% for scores ≤0.2. This indicates that patients with low genomic risk have a minimal likelihood of harboring aggressive disease, reinforcing the safety of surveillance in this population.

### Intermediate-Risk Prostate Cancer

For patients with intermediate-risk prostate cancer, treatment decisions often involve balancing the risk of disease progression with the potential morbidity of intensified therapy. The Decipher GC provides molecular insights that refine treatment selection, helping distinguish those who can be safely managed with RT alone from those who would benefit from treatment intensification by adding short-term ADT. Data from the NRG/RTOG 0126 Phase III randomized trial demonstrated that patients with a low GC score (≤0.45) had favorable long-term outcomes with RT alone, showing a 10-year distant metastasis (DM) rate of only 4%. In contrast, patients with a high GC score (≥0.60) had a 16% DM rate, suggesting that treatment intensification with short-term ADT may provide significant benefits in reducing metastasis risk.^22^ These insights highlight the growing role of molecular classifiers in tailoring therapy for intermediate-risk prostate cancer, ensuring a more individualized and evidence-based approach to treatment planning.

The NRG GU-010 (GUIDANCE, NCT05050084) trial is an ongoing Phase III study using Decipher GC scores to guide treatment de-intensification or intensification for unfavorable intermediate-risk prostate cancer.^28^ Patients with lower GC risk are randomized to RT alone or RT with short-term (6 months) ADT, assessing whether ADT can be safely omitted. Those with higher GC risk are randomized to RT with ADT or RT with ADT plus darolutamide, evaluating the benefits of intensified systemic therapy with an androgen receptor pathway antagonist. This trial aims to refine risk-adapted treatment strategies, minimizing unnecessary toxicity while ensuring high-risk patients receive adequate therapy, further reinforcing the role of GC in precision oncology.

### High-risk Prostate Cancer

For high-risk prostate cancer, the Decipher GC provides critical prognostic insights that help refine treatment intensification strategies. A meta-analysis of 3 Phase III randomized trials (NRG/RTOG 92-02, 94-13, and 99-02) demonstrated that the GC independently predicted key oncologic outcomes, including DM, BCR, and PCSM. Patients with high GC scores had a 10-year DM rate of 26%, significantly higher than the 6% observed in low-risk patients. The benefit of long-term ADT (24 months) over short-term ADT (4 months) was greatest in GC high-risk patients, reducing DM risk from 31% to 20%, while low-risk patients derived minimal additional benefit.^21^ These findings suggest that the GC can identify patients who would benefit most from intensified systemic therapy, guiding more personalized treatment decisions.

The NRG GU-009 (PREDICT-RT, NCT04513717) trial is an ongoing Phase III study designed to personalize treatment strategies for high-risk prostate cancer based on the Decipher GC.^29^ Patients with lower GCs are randomized to RT with either 12 or 24 months of ADT, evaluating whether shorter-duration ADT can maintain efficacy while reducing toxicity. Conversely, higher GC risk and/or node-positive patients are randomized to RT with 24 months of ADT alone or intensified treatment of RT with 24 months of ADT plus 24 months of apalutamide, assessing whether adding systemic therapy improves outcomes in this higher-risk population. By using the GC as an integral biomarker for trial randomization, NRG GU-009 aims to optimize risk-adapted treatment approaches, balancing treatment intensity with toxicity management in high-risk prostate cancer.

### Post-Radical Prostatectomy

For post-RP patients who have rising PSA levels, the Decipher GC provides important prognostic insights to guide salvage RT and potential incorporation of ADT. Data from 2 Phase III trials (RTOG 96-01, SAKK 09/10) demonstrated that GC high-risk patients (>0.60) had a significantly higher risk of biochemical progression, clinical progression, and DM, with greater benefit from the addition of ADT to salvage RT.^30^ In RTOG 96-01, GC high-risk patients receiving ADT had a 12-year reduction in DM (11.2%) and PCSM (8.4%), whereas GC low-risk patients derived minimal or even negative survival benefit from ADT.^31^ Similarly, SAKK 09/10 found that GC high-risk patients had higher rates of salvage ADT use after RT compared to low-risk patients. Given these findings, using the GC could individualize treatment decisions, particularly in determining which patients should receive ADT alongside salvage RT, and identify those who would benefit most from early intervention.^32^^,33^ However, it is important to note that the optimal duration of ADT (e.g., 6 vs. 24 months) based on Decipher GC risk stratification remains unknown.

### Challenges and Limitations

While the 22-gene GC has shown significant promise in refining risk stratification and guiding treatment decisions, several challenges and limitations remain. One major concern is the heterogeneity in study designs, as much of the supporting evidence comes from retrospective analyses. While these studies have demonstrated the GC’s prognostic value across various clinical settings, the lack of prospective, randomized validation in some treatment scenarios limits the generalizability of findings. Differences in patient populations, treatment protocols, and follow-up durations across studies further contribute to variability in reported outcomes, making it difficult to establish uniform clinical guidelines based on the GC results alone. Additionally, inherent heterogeneity in prostate biopsy samples—including tumor multifocality and sampling variability—may affect GC scoring and interpretation. The integration of novel imaging modalities, such as PSMA PET, may help refine risk stratification by improving tumor characterization and guiding targeted biopsy approaches. Another key limitation is that the GC primarily functions as a prognostic rather than a predictive biomarker. While it provides valuable risk stratification by estimating the likelihood of metastasis, BCR, or PCSM, it does not directly predict treatment response.

Despite these challenges, ongoing large, randomized prospective trials, such as NRG GU-009 and NRG GU-010, aim to address these limitations by evaluating the clinical utility of the GC in real-time treatment decision-making. These trials will provide high-level evidence on how genomic risk stratification can guide treatment de-intensification or intensification, ultimately helping to bridge the gap between prognostic insights and actionable predictive biomarkers. As results from these studies become available, they will further clarify the GC’s role in optimizing prostate cancer management and shaping future clinical guidelines.

## Conclusion

The integration of the Decipher GC into prostate cancer management represents a significant advancement in personalized risk stratification and treatment decision-making. By providing independent prognostic information beyond traditional clinical factors, the GC enhances the ability to distinguish which patients may benefit from treatment escalation and which may be safely managed with de-intensified approaches. The results of ongoing prospective clinical trials, such as NRG GU-009 and NRG GU-010, are expected to further solidify the clinical utility of the GC in real-time treatment selection. Moving forward, the continued evolution of molecular diagnostics, combined with emerging therapeutic advancements, will further enhance precision oncology in prostate cancer, ensuring that patients receive the most appropriate, individualized care.

## Data Availability

The data that support the findings of this study are available on request from the corresponding author.
